# Low-temperature growth of highly crystalline β-Ga_2_O_3_ nanowires by solid-source chemical vapor deposition

**DOI:** 10.1186/1556-276X-9-347

**Published:** 2014-07-10

**Authors:** Ning Han, Fengyun Wang, Zaixing Yang, SenPo Yip, Guofa Dong, Hao Lin, Ming Fang, TakFu Hung, Johnny C Ho

**Affiliations:** 1 Department of Physics and Materials Science, City University of Hong Kong, 83 Tat Chee Ave., Hongkong SAR, People's Republic of China; 2 Cultivation Base for State Key Laboratory, Qingdao University, No. 308 Ningxia Road, Qingdao 266071, People's Republic of China; 3 Shenzhen Research Institute, City University of Hong Kong, Shenzhen 518057, People's Republic of China

**Keywords:** β-Ga_2_O_3_ nanowires, Chemical vapor deposition, Solid-source, Highly crystalline, Large resistance, Dielectric

## Abstract

Growing Ga_2_O_3_ dielectric materials at a moderately low temperature is important for the further development of high-mobility III-V semiconductor-based nanoelectronics. Here, β-Ga_2_O_3_ nanowires are successfully synthesized at a relatively low temperature of 610°C by solid-source chemical vapor deposition employing GaAs powders as the source material, which is in a distinct contrast to the typical synthesis temperature of above 1,000°C as reported by other methods. In this work, the prepared β-Ga_2_O_3_ nanowires are mainly composed of Ga and O elements with an atomic ratio of approximately 2:3. Importantly, they are highly crystalline in the monoclinic structure with varied growth orientations in low-index planes. The bandgap of the β-Ga_2_O_3_ nanowires is determined to be 251 nm (approximately 4.94 eV), in good accordance with the literature. Also, electrical characterization reveals that the individual nanowire has a resistivity of up to 8.5 × 10^7^ Ω cm, when fabricated in the configuration of parallel arrays, further indicating the promise of growing these highly insulating Ga_2_O_3_ materials in this III-V nanowire-compatible growth condition.

**PACS:**

77.55.D; 61.46.Km; 78.40.Fy

## Background

In the past decade, gallium oxide (Ga_2_O_3_), as a large-bandgap (approximately 4.9 eV) semiconductor, has attracted extensive attention in the area of insulating oxides for the metal-oxide-semiconductor (MOS) technology as well as the active materials for the solar-blind deep ultraviolet detectors [1-6]. In particular, when high-mobility III-V compound semiconductor nanomaterials, such as GaAs, InAs, GaSb, and InSb nanowires (NWs), have been successfully illustrated with their great technological potentials in next-generation electronics [7-9], Ga_2_O_3_-based gate dielectrics are of significant importance to be achieved and to outperform the conventional silicon technology, due to their excellent stability and relatively high dielectric constant (approximately 14.2) as compared to that of SiO_2_ (approximately 3.9) or even the typically used high-κ Al_2_O_3_ (approximately 8) [1,10].

Till now, there are several effective integrations of Ga_2_O_3_-based gate dielectrics demonstrated in thin-film III-V field-effect transistors (FETs). For instance, Ga_2_O_3_ and Ga_2_O_3_/Gd_2_O_3_ composite materials have been shown to be excellent gate dielectrics for GaAs, In_*x*_Ga_1 − *x*_As, and GaN thin-film transistors with the low interface state density and high breakdown field strength [2,3,7,11]. However, there are still very few studies focused on Ga_2_O_3_ dielectrics prepared directly on III-V NWs since the typical thermal oxidizing method is challenging to be executed on the small-diameter NWs, while the atomic-layer-deposited (ALD) high-κ HfO_2_ and Al_2_O_3_ dielectrics often have significant interfacial defects when performed on NW materials [12]. In this case, it is necessary to explore other alternative dielectrics such as Ga_2_O_3_ achieved by other advanced techniques in order to tackle this issue for the versatile high-mobility III-V NW devices.

Among many Ga_2_O_3_ phases, the monoclinic β-Ga_2_O_3_ is the most stable phase, being a promising gate dielectric alternative; nevertheless, it often requires synthesis at high temperatures to maintain its excellent crystallinity. For example, β-Ga_2_O_3_ NWs are usually prepared at above 1,000°C, employing Ga metal as the source in the chemical vapor deposition (CVD) [13], and sometimes even high-energy arc plasma is utilized when using GaN as the starting material [14]. As most III-V NWs are synthesized at a moderate temperature in the range 400°C to 600°C via vapor-liquid-solid (VLS) and/or vapor-solid-solid (VSS) mechanisms [15-18], a compatible low-temperature β-Ga_2_O_3_ growth technique is therefore essential to grow dielectrics laterally on III-V NWs while not degrading the III-V NW materials with high vapor pressures.

Recently, we have adopted various III-V material powders as precursor sources for the NW growth by CVD, such as obtaining GaAs, InP, GaSb, etc. at a temperature of 500°C to 600°C [19-21]. Here, in this report, we perform detailed studies on the synthesis behaviors and fundamental physical properties of β-Ga_2_O_3_ NWs at this moderate growth temperature in a similar CVD growth system. It is revealed that highly crystalline and insulating β-Ga_2_O_3_ NWs are successfully grown on the amorphous SiO_2_ substrate, which provides a preliminary understanding of the β-Ga_2_O_3_ NWs attained by the solid-source CVD method, and further enables us to manipulate the process parameters to achieve high-quality gate dielectrics laterally grown on III-V semiconductor NWs for the coaxially gated NW device structures [22].

## Methods

### Synthesis of Ga_2_O_3_ NWs

The Ga_2_O_3_ NWs were synthesized in a dual-zone horizontal tube furnace, where the upstream zone was used for evaporating the solid source and the downstream zone for the NW growth, as reported previously [15]. At first, 50-nm Au colloids (standard deviation of approximately 5 nm, NanoSeedz, Hong Kong) were drop-casted on SiO_2_/Si substrates (50-nm thermally grown oxide) to serve as the catalyst, which were then placed in the middle of the downstream zone with a tilt angle of approximately 20°. The solid source, GaAs powders (approximately 1.0 g), was contained in a boron nitride crucible, which was then positioned in the upstream zone with a distance of approximately 10 cm away from the substrate with catalysts. During the NW growth, the substrate was initially heated to the preset growth temperature (580°C to 620°C) and the source was then heated to the required source temperature (900°C). Mixture of argon (Ar, 99.9995% purity, 100 sccm) and oxygen (O_2_, 99.9995% purity) in different flow ratios (100:1 to 100:100) was used as the carrier gas to transport the thermally vaporized precursors to the downstream. After the growth of 1 h, the source and substrate heater were stopped together and cooled down to room temperature under the Ar and O_2_ flow.

### Characterization of Ga_2_O_3_ NWs

Surface morphologies of the grown Ga_2_O_3_ NWs were examined with a scanning electron microscope (SEM; FEI/Philips XL30, Hillsboro, OR, USA) and transmission electron microscope (TEM; Philips CM-20, Amsterdam, The Netherlands). Crystal structures were determined by collecting X-ray diffraction (XRD) patterns on a Philips powder diffractometer using Cu Kα radiation (*λ* = 1.5406 Å) and by selected area electron diffraction (SAED; Philips CM-20). Elemental analysis was performed using an energy-dispersive X-ray (EDS) detector attached to JEOL CM-20 (Akishima-shi, Japan) to measure the chemical composition of the grown NWs. For the TEM and EDS analyses, the Ga_2_O_3_ NWs were first suspended in an ethanol solution by ultrasonication and drop-casted onto a copper grid for the corresponding characterization. The reflectance spectrum was measured with a LAMBDA 750 spectrophotometer (PerkinElmer, Waltham, MA, USA) at room temperature.

The Ga_2_O_3_ NW arrays were fabricated by contact printing on SiO_2_/Si substrates (50-nm thermally grown oxide) as reported previously [23]. Typically, a pre-patterned SiO_2_/Si substrate coated with a photoresist was used as the receiver, while the donor NW chip was flipped onto the receiver and slid at a rate of 10 mm/min with a pressure of 50 g/cm^2^. After photoresist removal, the Ga_2_O_3_ NW arrays were left on the patterned region. Then, photolithography was utilized to define the electrode regions, and a 100-nm-thick Ni film was thermally deposited as the contact electrode followed by a lift-off process. The electrical performance of the fabricated NW arrays was characterized with a standard electrical probe station and Agilent 4155C semiconductor analyzer (Santa Clara, CA, USA).

## Results and discussion

**Figure 1:**
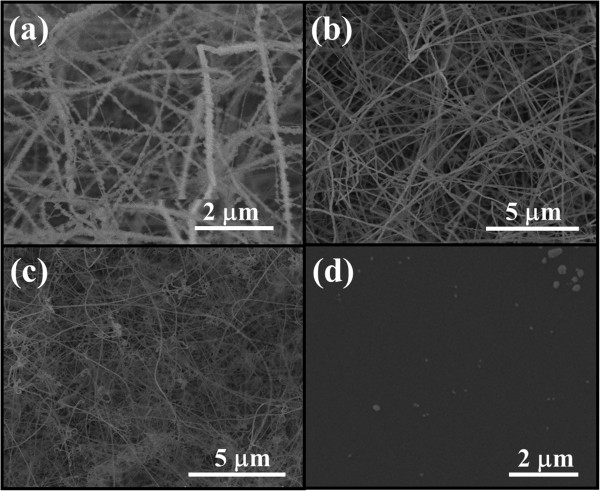
**SEM images of the Ga**_**2**_**O**_**3 **_**NWs grown at different Ar:O**_**2 **_**flow ratios.** Source temperature at 900°C, substrate temperature at 610°C, Ar flow of 100 sccm. **(a)** 100:1. **(b)** 100:2. **(c)** 100:10. **(d)** 100:100.

As reported previously, we synthesized GaAs NWs by the solid-source CVD method using GaAs powders as the source material heated at 900°C and 100-sccm H_2_ as the carrier gas, catalyzed by Au nanoparticles at 580°C to 620°C [15,24]. In an attempt to prepare Ga_2_O_3_ in a compatible circumstance, we employ the same conditions here except the H_2_ carrier gas, which is substituted by a mixture of Ar and O_2_ in order to introduce oxygen into the growth environment. The flow rate of Ar is fixed at 100 sccm, and the ratio of Ar and O_2_ is precisely tailored as 100:1, 100:2, 100:10, and 100:100 by using mass flow controllers (the pressure is approximately 0.8, approximately 0.8, approximately 0.9, and approximately 1.4 Torr, respectively). The corresponding obtained NW products appeared whitish on the substrate, in contrast with the yellowish-green GaAs NWs. The NWs are then observed by SEM as shown in Figure 1a,b,c,d. It is clear that the NWs grown at the Ar:O_2_ flow ratio of 100:2 are relatively long and smooth on the surface (Figure 1b), while the lower O_2_ flow induces a significant coating problem (Figure 1a) and the higher O_2_ flow suppresses the NW growth (Figure 1c,d). The high O_2_ flow might deactivate the Au catalyst leading to no NW growth, while the low O_2_ flow might not make the Ga_2_O_3_ NW nucleation sufficient over the GaAs NW growth but only overcoat on the GaAs NW surface resulting in the overcoating problem. Notably, in our former study of GaAs NWs, the GaAs powder source has depleted less than 0.1 g of weight after the growth, whereas the source has now depleted more than 0.5 g of weight in this Ga_2_O_3_ NW growth by introducing a small amount of oxygen. This would be attributed to the fact that even though Ga has a decently high vapor pressure, there is still a small amount of Ga being evaporated and transported in the H_2_ atmosphere in the GaAs NW growth. On the other hand, when O_2_ is introduced in the Ga_2_O_3_ NW growth, Ga is easily oxidized to Ga_2_O [25], which has a far higher vapor pressure than that of metallic Ga, and thus can be massively evaporated and transported by the carrier gas to the substrate; as a result, a proper control in the amount of O_2_ feed is critical for the effective NW growth here.

**Figure 2:**
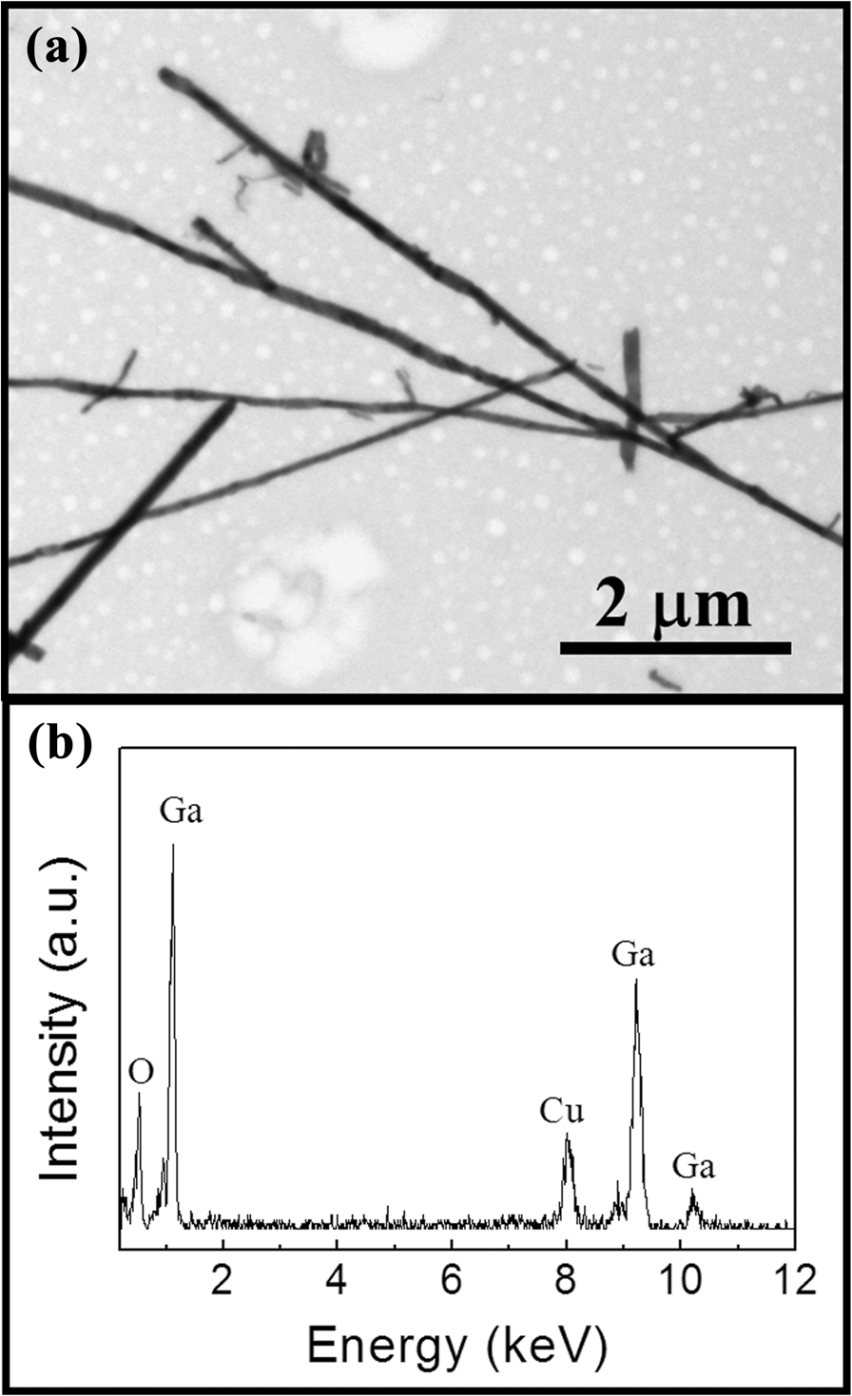
**Morphology and elemental analysis of the β-Ga**_**2**_**O**_**3 **_**NWs grown at the Ar:O**_**2 **_**flow ratio of 100:2. (a)** TEM image. **(b)** EDS spectrum.

The NWs grown at the Ar:O_2_ flow ratio of 100:2 are then observed by TEM as depicted in Figure 2a, which further confirms the straight NWs with smooth surfaces. Furthermore, the elemental composition is analyzed by EDS, and the typical spectrum is illustrated in Figure 2b, which clearly demonstrates that the NWs are mainly composed of Ga and O with an atomic ratio of approximately 2:3. These results evidently show that the obtained NWs here are Ga_2_O_3_ instead of the GaAs NWs grown in the H_2_ atmosphere. It should also be noted that although As-doped In_2_O_3_ NWs were prepared in a similar system when utilizing InAs powders as the source material and As is detected in the EDS spectrum [26], no As-related signal is obtained within the detection limit of EDS performed in this study. This difference may be due to the alteration in the synthesis condition that H_2_ is intentionally introduced into the Ar/O_2_ carrier gas to suppress the oxide growth in [25], which can be ruled out in this Ga_2_O_3_ NW growth. It is plausible that since oxygen has a far higher electron negativity (approximately 3.44) than arsenic (approximately 2.18) and that Ga_2_O_3_ has a far lower Gibbs free energy (approximately −998.3 kJ/mol) than GaAs (−67.8 kJ/mol) [27], in this case, Ga_2_O_3_ is more preferentially grown from the thermal dynamics point of view. In other words, when H_2_ in introduced, Ga_2_O_3_ growth would be deterred and get substituted by the GaAs growth [25].

**Figure 3:**
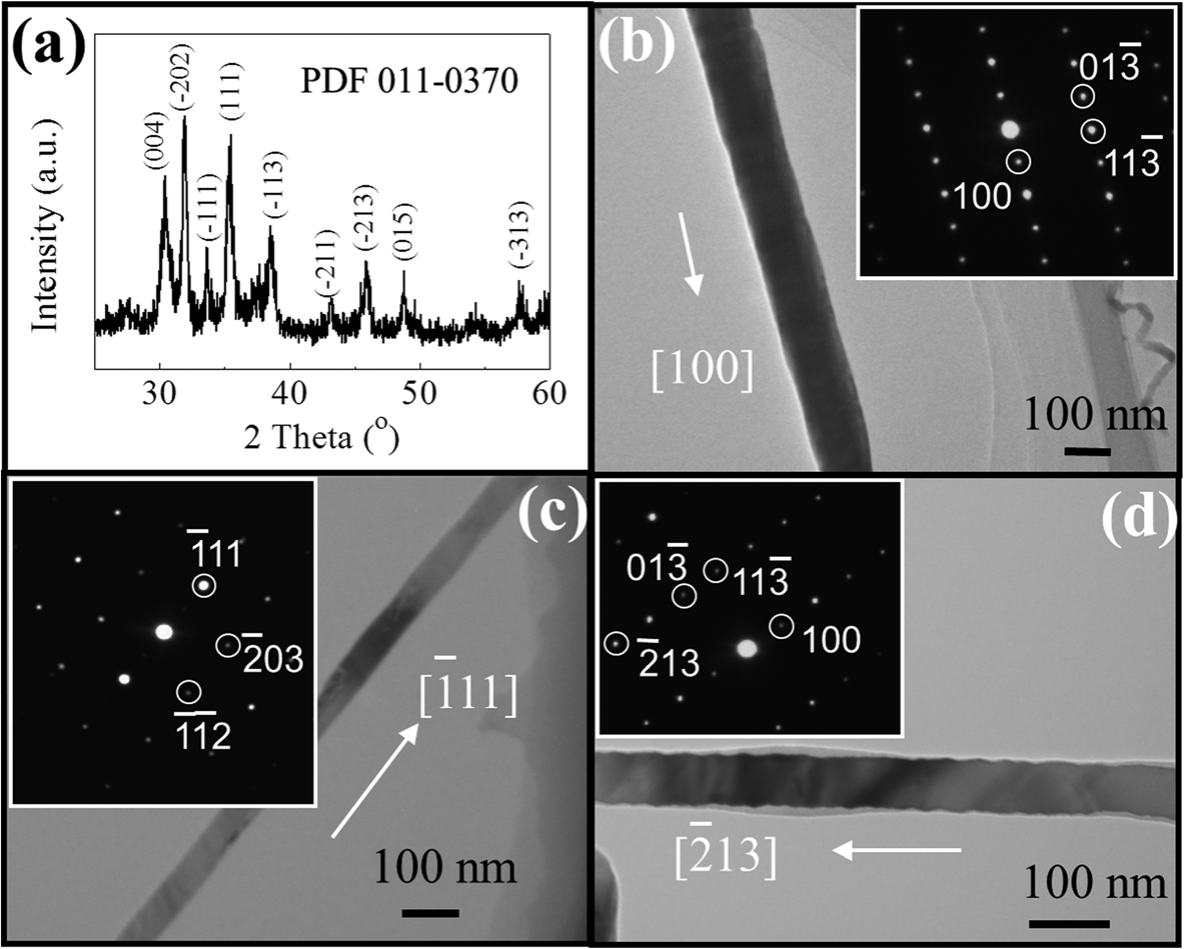
**Structural and orientation analysis of the β-Ga**_**2**_**O**_**3 **_**NWs grown at the Ar:O**_**2 **_**flow ratio of 100:2. (a)** XRD pattern. **(b, c, d)** TEM images and the corresponding SAED patterns (insets).

In order to investigate the crystal structure of the obtained Ga_2_O_3_ NWs, the XRD pattern is attained for NWs readily grown on the SiO_2_/Si substrate as presented in Figure 3a. It is obvious that the NWs are grown in the monoclinic structure (β-phase) in accordance with the standard card PDF 011-0370. Then, the crystal structure and growth orientation of individual NWs are further studied by using SAED as shown in Figure 3b,c,d. All these indicate that the representative NWs all existed in the monoclinic crystal structure, which is in good agreement with the XRD results. Even though the orientations are observed to vary from NW to NW, typically low-index directions such as [100], $\left[\bar{1}11\right]$, and $\left[\bar{2}13\right]$ are perceived, which might have resulted from the similar surface energies of these crystal planes, especially for materials in the nanometer size with the examples reported in Si NWs [28], GaAs NWs [15], ZnSe NWs [29], etc.

**Figure 4:**
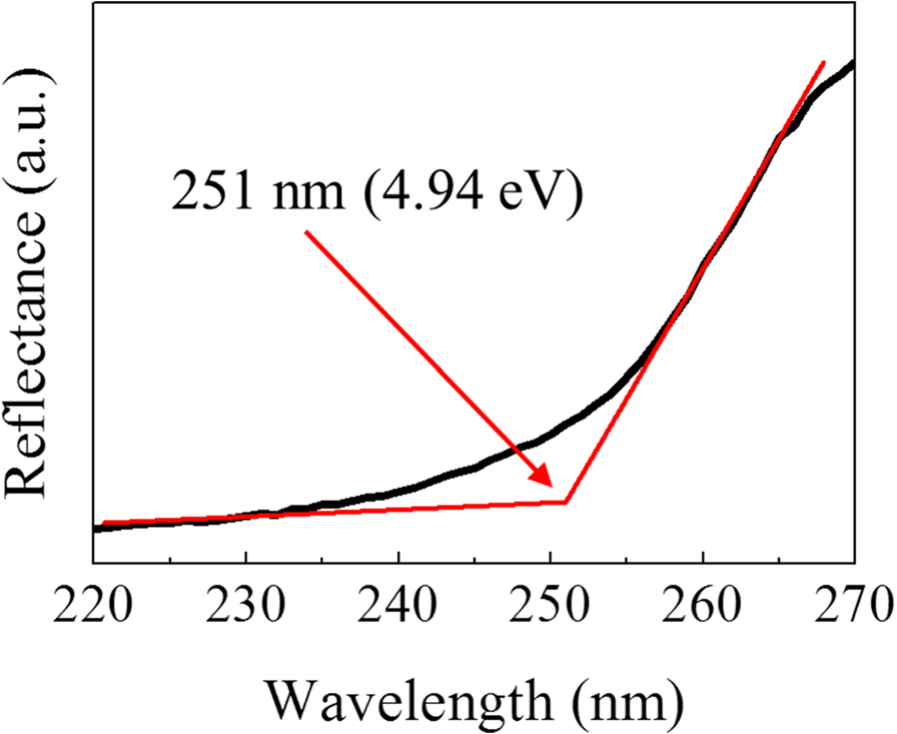
**Reflectance spectrum of the β-Ga**_**2**_**O**_**3 **_**NWs grown at the Ar:O**_**2 **_**flow ratio of 100:2.**

The bandgap of β-Ga_2_O_3_ NWs can also be determined by the reflectance spectrum as depicted in Figure 4. It clearly shows that the absorption edge lies at approximately 251 nm (4.94 eV). This bandgap value is in good agreement with that of β-Ga_2_O_3_ NWs reported in the literature (approximately 254 nm) [30] while a bit higher than that of bulk materials (approximately 270 nm) [31]. A relatively larger bandgap of nanomaterials is often observed than their bulk counterparts, which is usually attributed to the quantum confinement effect of nanomaterials, inducing a blueshift of the bandgap [32].

**Figure 5:**
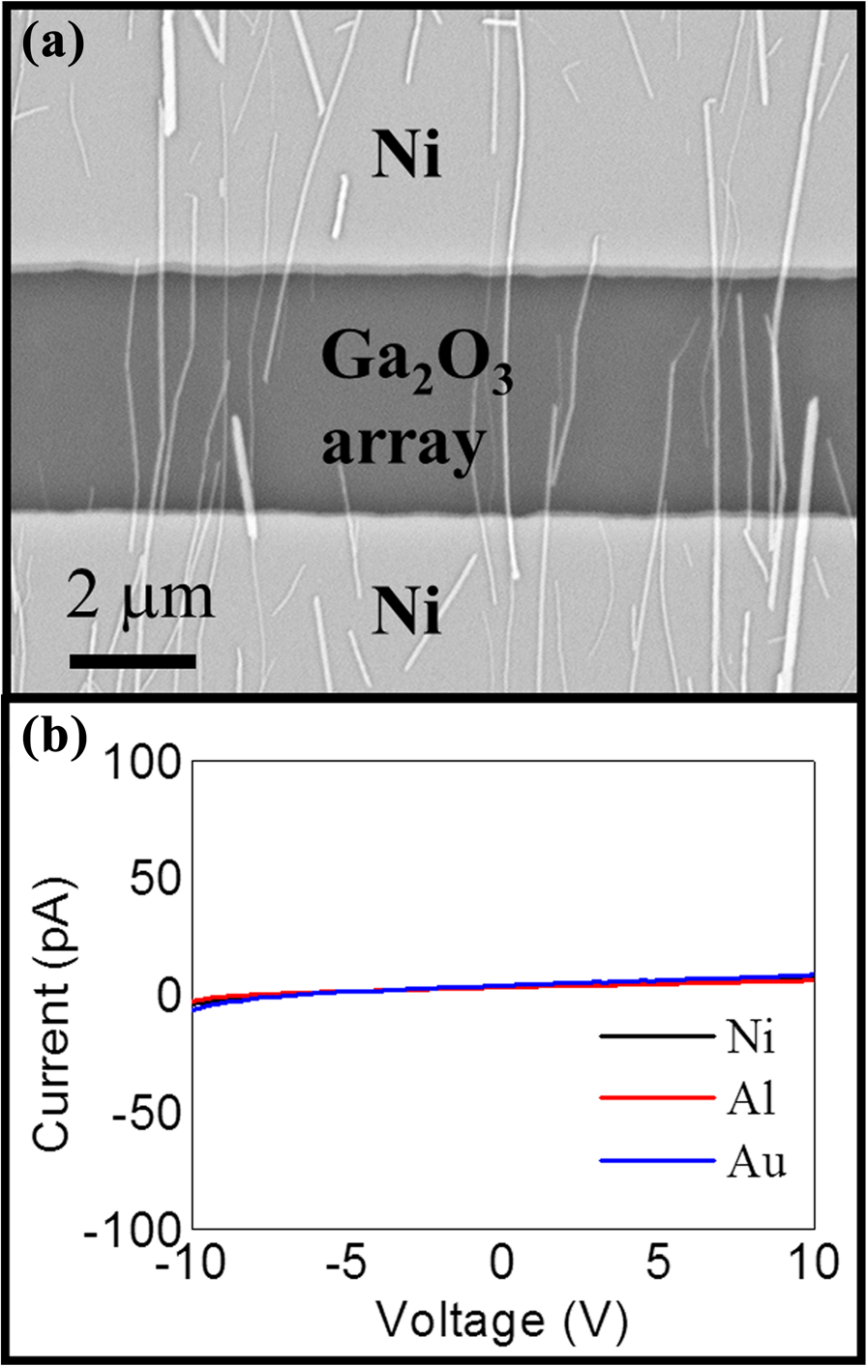
**Electrical properties of the β-Ga**_**2**_**O**_**3 **_**NWs grown at the Ar:O**_**2 **_**flow ratio of 100:2. (a)** SEM image of the printed β-Ga_2_O_3_ NW arrays patterned with Ni electrodes on both ends. **(b)** The corresponding *I*-*V* curve of the β-Ga_2_O_3_ NW arrays with Ni, Al, and Au as electrodes.

To shed light on exploring the electronic properties of achieved β-Ga_2_O_3_ NWs, the resistance of NWs is first assessed by defining electrodes by standard photolithography. It should be noted that when defining Ni electrodes on a single β-Ga_2_O_3_ NW, no significant current can be obtained as compared with the resolution (approximately 1 pA) of our semiconductor analyzer and probe station. In order to enlarge the current signal to a measurable level, the β-Ga_2_O_3_ NWs are then aligned into parallel arrays by the contact printing technique as reported previously [8,23]. Ni electrodes (with the work function of approximately 5.1 eV) are then defined on both ends of the NW arrays, given in the SEM image in Figure 5a. The NW density is approximately 1 NW/μm, accounting for approximately 200 NWs in the 200 μm (width) × 3.7 μm (length) channel area. In this way, the current-voltage (*I*-*V*) curve of the representative β-Ga_2_O_3_ NW array is measured and shown in Figure 5b, where the resistance is estimated to be approximately 2 × 10^12^ Ω as the current is approximately 5 pA under 10-V bias. As a result, the resistance is approximately 4 × 10^14^ Ω per individual NW (approximately 2 × 10^12^ × 200 Ω, as 200 NWs are connected in parallel). Then, the resistivity can be estimated as 2 × 10^12^ × 200 Ω × 3.7 μm/3.14/50^2^ nm^2^ = 8.5 × 10^7^ Ω cm, considering the NW diameter of approximately 100 nm. Notably, other metal electrodes with different work functions such as Al (approximately 4.2 eV) and Au (approximately 5.3 eV) are also prepared, in which the results attained are all similar as shown in Figure 5b, suggesting the highly insulating property of the NWs here. This resistivity is relatively larger than those of doped and undoped β-Ga_2_O_3_ NWs reported in the literature [4,6,13], which can be attributed to the moderate growth temperature employed in this work such that less impurity would be incorporated, showing its prospective in dielectric materials for advanced III-V nanowire-based nanoelectronics.

## Conclusions

Highly crystalline β-Ga_2_O_3_ NWs are synthesized by a solid-source chemical vapor deposition method employing GaAs powders as the source material and mixture of Ar and O_2_ as the carrier gas. The NWs grown at the Ar:O_2_ flow ratio of 100:2 are long (>10 μm) with a uniform diameter of approximately 100 nm and smooth surfaces. X-ray diffraction and selected area electron diffraction results confirm the monoclinic structure of the obtained NWs with varied growth orientations along the low-index planes. Furthermore, the reflectance spectrum demonstrates the bandgap of β-Ga_2_O_3_ NWs being 4.94 eV, while the electrical measurement deduces the corresponding resistivity of 8.5 × 10^7^ Ω cm. All these results indicate the successful synthesis of a large-bandgap Ga_2_O_3_ material in III-V-compatible growth conditions, illustrating the promising potential for dielectric materials used for III-V nanowire-based metal-oxide-semiconductor technology.

## Competing interests

The authors declare that they have no competing interests.

## Authors’ contributions

NH synthesized the Ga_2_O_3_ NWs and drafted the manuscript. FW made the SEM and TEM observations, ZY carried out the XRD measurement, and SY carried out the reflectance spectrum. GD fabricated the NW array devices, and HL made the *I*-*V* measurement. MF made the SAED identification, and TH carried out the EDS spectrum. JCH provided the idea and completed the manuscript. All authors read and approved the final manuscript.
